# Supplementary figures and images for: Gene co-expression network analysis of the human gut commensal bacterium Faecalibacterium prausnitzii in R-Shiny
Source: PLoS One. 2022 Nov 18;17(11):e0271847. doi: 10.1371/journal.pone.0271847 (PMC9674144; doi:10.1371/journal.pone.0271847)

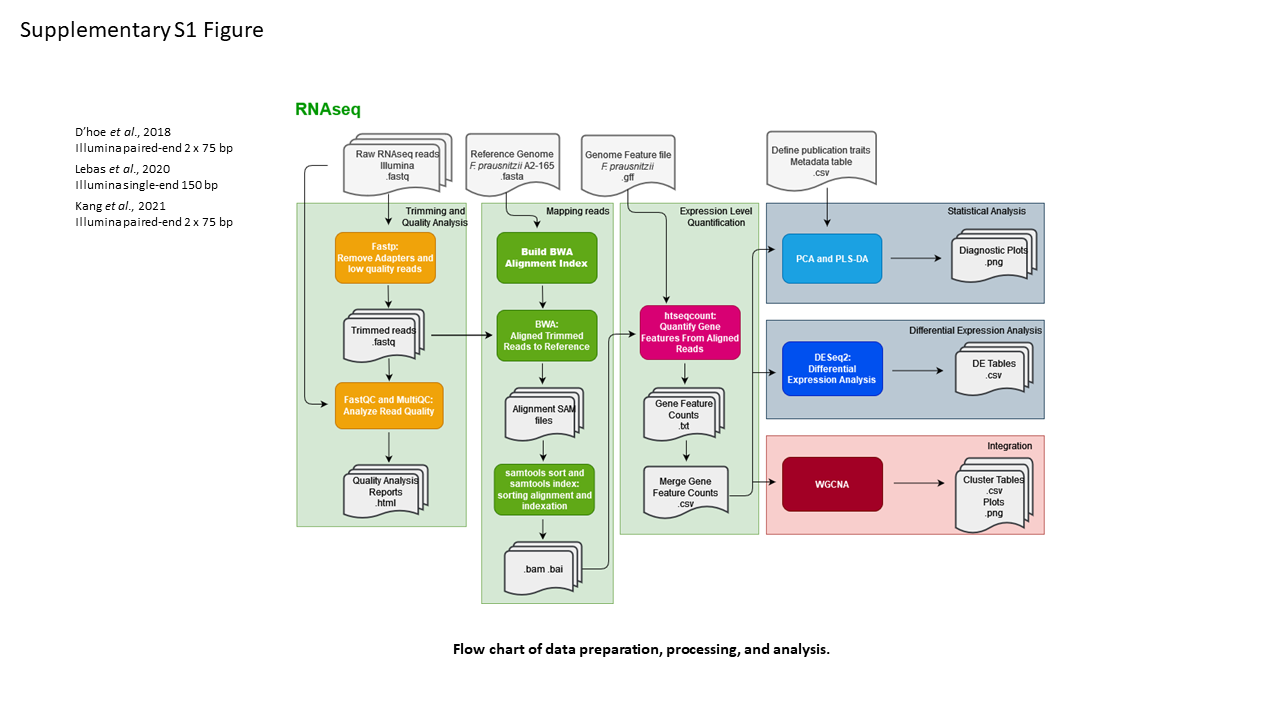

Supplement: S1 Fig — (TIF) [file pone.0271847.s001.tif]

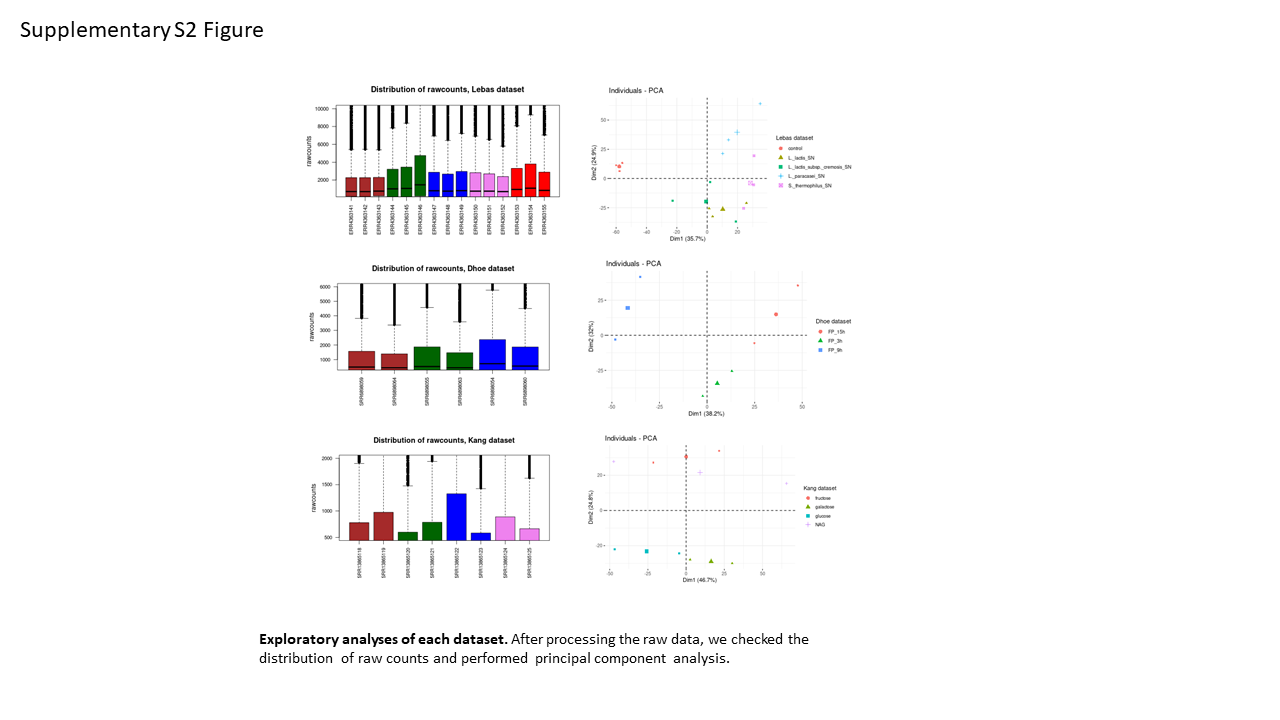

Supplement: S2 Fig — After processing the raw data, we checked the distribution of raw counts and performed principal component analysis. (TIF) [file pone.0271847.s002.tif]

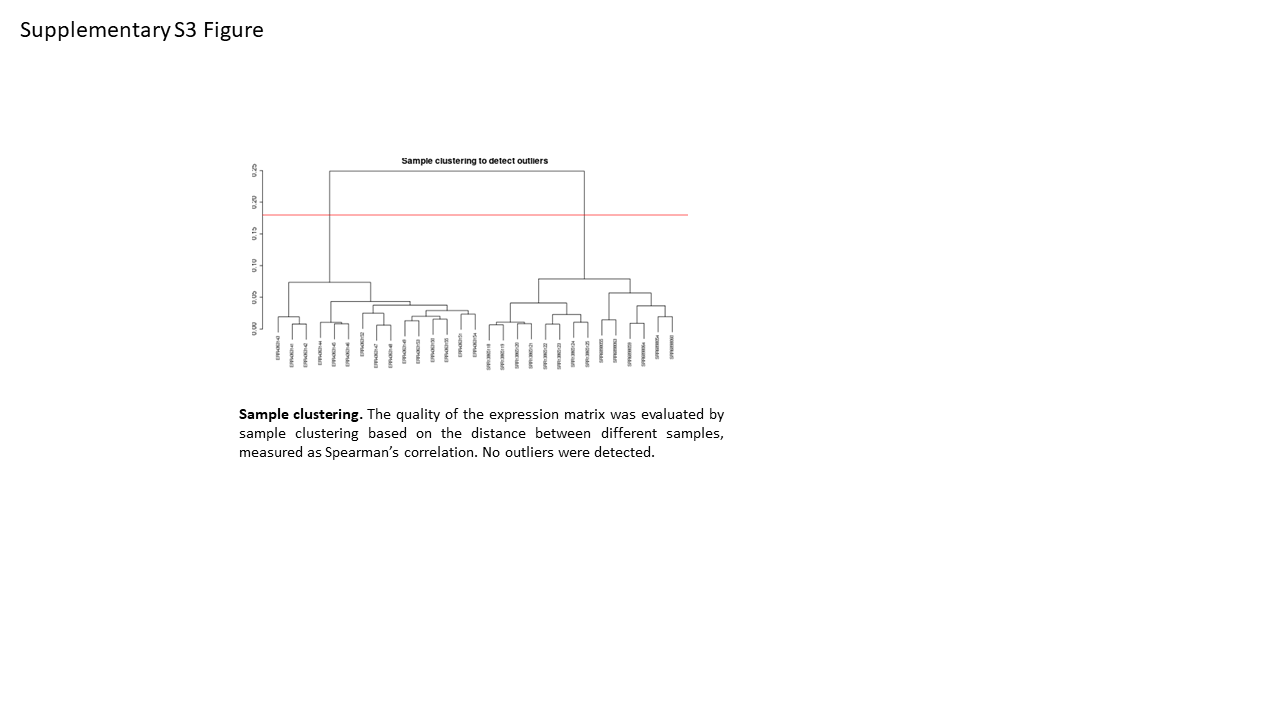

Supplement: S3 Fig — The quality of the expression matrix was evaluated by sample clustering based on the distance between different samples, measured as Spearman’s correlation. No outliers were detected. (TIF) [file pone.0271847.s003.tif]
