# Supplementary material for: Gene co-expression network analysis of the human gut commensal bacterium Faecalibacterium prausnitzii in R-Shiny
Source: PLoS One. 2022 Nov 18;17(11):e0271847. doi: 10.1371/journal.pone.0271847 (PMC9674144; doi:10.1371/journal.pone.0271847)
Supplement: Attachment — Submitted filename: Response to Reviewers.docx [file pone.0271847.s007.docx]

**Reviewer #1**

1. In the introduction, the authors mention the idea of using F. prausnitzii as a "real" probiotic. However, the results of this work indicate that Faecalibacterium prausnitzii alone might not be sufficient in case it could be uptaken as a probiotic since its functionality seems to be highly dependent on environmental conditions. If this is true, it would be interesting to add a comment regarding this point of view, considering that this probiotic would be given to patients with sometimes heavily altered ecosystems.

We agree with Reviewer#1 who raises a sensitive point on the use of F. prausnitzii as a probiotic and even on the use of probiotics in general. We added a comment in the Conclusion section:

“As *F. prausnitzii* is considered a promising next generation probiotic, our results raise the question of its functionality, which seems related to environmental conditions. This aspect must be taken into account for the optimization in the administration of *F. prausnitzii* as a probiotic in patients with an altered digestive ecosystem.”

2. The provided website (https://feaprau.omics.ovh/) for data exploration does not work. Will it be available after the publication of the paper?

We apologize for the inconvenience. Now the site is in public access. It is accessible at the following link: <https://faeprau.omics.ovh/>.

3. ¿Is there any relationship between the outcome of the analysis and the composition of the culture media used?

In the three studies, the media used were rich media whose composition was partially defined. They all contained broths of peptones, casa amino acids, or yeast extracts. They were supplemented with carbon sources, vitamins and/or short chain fatty acids. We tested if there was a relationship between the modules defined by WGCNA and the carbon source used in the different samples. In addition, we also tested whether there was a relationship between the modules and the supplementation of short-chain fatty acids in the medium. However, we did not find any relationship between the outcome of the analysis and the carbon sources or the short-chain fatty acids present in the culture media.

We believe that more RNAseq data are necessary to highlight precise relationships between the outcomes and the composition of the culture medium. We hope to enrich and update our analyses with future available RNAseq data.

4. Line 209: "are to known to function"; delete first "to"

Done

5. Line 276: "have important functions in certain conditions.", consider "...under certain conditions".

Done

**Reviewer #2**

1. Consider in the title to develop WGCNA acronym or rephrase to avoid its use.

We reconsidered the title to avoid the use of the method WGCNA: “Gene co-expression network analysis of the human gut commensal bacterium Faecalibacterium prausnitzii in R-Shiny”.

2. L92: MAM acronym should be placed after its definition.

Done

3. In general, figures were difficult to see clearly. Please, consider to upload an improved version for all of them (main figures and those in supplementary material) with better resolution.

We have reviewed the resolution of the figures.

4. Additional points to be discussed/comment further:

• It is not clear to me if in the future, it will be possible to include in the tool developed additional data of RNA seq studies that will arise from the same strain in other conditions or from other F. prausnitzii strains. Please, discuss further on this possibility.

Indeed, we have developed this tool with the aim of making it evolve by adding future public RNAseq data from other research teams studying the A2-165 strain.

We are studying two possibilities:

(1) the researchers will upload their data in a transitory way on the interface, but there is a risk of bias in normalizing the data.

(2) they will contact the corresponding authors in order to normalize on all the datasets, and so that their data is permanently added to the interface.

For now, we have focused on the strain A2-165 as it is the only strain for which there is RNAseq data. As suggested by Reviewer #2, we can consider integrating RNAseq data from other strains in the future.

We added a sentence at this end of the discussion section: “In the future, this interface may evolve in order to add RNAseq data generated by research teams studying F. prausnitzii.”

• L195-197 the authors evidence that the gene expression datasets cluster apart. Table 1 evidences that a different number of samples (and therefore reads) have been included in the analysis for each study. However, all are analysed together in the WGCNA construction. I wonder if there could be a bias due to this differences in number of samples/reads. How could this have influenced in the weighted gene co-expression network? (for example that the network modules are more representative of Lebas study). If the authors have performed any test to control this is not taking place, please provide further details.

WGCNA is an unsupervised method, which analyzes and aggregates unlabeled datasets. Thus, the difference in number of samples in each datasets does not influence the construction of the co-expression networks. As the number of reads is different from one sample to another, we applied a normalization step to take these differences into account.

In Figure 1A, the PLSDA showed that the Lebas dataset is strongly separated from the other two datasets. In addition, we also observed by principal component analysis, which ignores the information regarding the class of the samples, that the samples from the Lebas dataset are also strongly separated from the others. It is therefore not surprising that certain genes in the modules are only co-expressed in the Lebas dataset.

The PCA can be viewed at the following link: <https://faeprau.omics.ovh/>.

• In the identification of hub and bottleneck genes a threshold value has been used (>210 degree of connectivity and >300 betweenness, respectively). As far as I understood, no correction per number of genes in the module has been carried out. I suggest to add an analysis considering this additional parameter because in modules with a limited number of genes connectivity among them could be also high, but not reaching the thresholds set simply because they are smaller modules. Then the % of connectivity/betweenness considering the number of genes in the module could be evaluated to identify genes of interest.

We thank Rewiever #2 for this very interesting remark. In gene co-expression network, hub genes (with high degree of connectivity) interact with many other genes. On other hand, the measure of betweenness is based on the theory that important genes lie on many paths between other genes in the network. In this context, we were interested in the genes with the highest values of connectivity and/or betwenness because they are likely to be biologically important for the pathways or processes represented by a particular module.

We agree with Rewiever#2 that modules containing fewer genes could also have strongly connected genes. Thus, we looked at the measures of connectivity and betweeness within each module without applying an absolute hub/bottleneck threshold but by using a relative hub/bottleneck threshold of top 10%. As a result, we detected only one gene in the module lightyellow (12 genes in total) that could be considered as a hub and/or a bottleneck (with a degree of connectivity = 10 and a betweenness = 45). And we detected only 3 hub genes (degree of connectivity = 17) and 4 bottleneck genes (betweenness > 31) in the module grey (27 genes in total). These 7 genes are located in the same operon. Thus, we are not sure of the relevance of this observation. More RNAseq experiments may lead to more robust and refined results. We hope to enrich and update our analyses with future available RNAseq data.

For this reviewer it has not been possible to fully evaluate the utility of the tool as it was not publicly available. I suggest for future submissions to consider implementing an option of access for those who have to review the work.

We apologize for the inconvenience. Now the site is in public access. It is accessible at the following link: <https://faeprau.omics.ovh/>.
